# Adopting common data elements for the National Trauma Research Repository through a consensus meeting: the trauma core

**DOI:** 10.1136/tsaco-2025-002064

**Published:** 2026-07-02

**Authors:** John David Cull, Pamela J Bixby, Nee-Kofi Mould-Millman, Smith Foster Heavner, Nicolas W Medrano, Olga Volk, Suresh Agarwal, Jennifer M Gurney, Mary J Homer, Avery B Nathens, James Boyce Phillips, Travis Polk, Michelle A Price, Carl I Schulman, Kevin M Schuster, Deborah M Stein, Shelly Timmons, Nathan White, The NTRR Investigator Group, Ashley N Moreno

**Affiliations:** 1 Surgery, Prisma Health Upstate, Greenville, South Carolina, USA; 2 Coalition for National Trauma Research, San Antonio, Texas, USA; 3 Emergency Medicine, University of Colorado Anschutz Medical Campus, Aurora, Colorado, USA; 4 Society of Critical Care Medicine, Mount Prospect, Illinois, USA; 5 Biomedical Sciences, Critical Path Institute, Tucson, Arizona, USA; 6 BRICS, Publicis Sapient, Boston, Massachusetts, USA; 7 Duke University Medical Center, Durham, North Carolina, USA; 8 Defense Committees on Trauma, Joint Trauma System, JBSA Fort Sam Houston, Texas, USA; 9 Department of Surgery, San Antonio Military Health System, San Antonio, Texas, USA; 10 US Department of Health and Human Services, Washington, District of Columbia, USA; 11 American College of Surgeons, Chicago, Illinois, USA; 12 Defense Health Agency, US Department of Defense, Washington, District of Columbia, USA; 13 Surgery, University of Pittsburgh, Pittsburgh, Pennsylvania, USA; 14 Center for Military Medicine Research, University of Pittsburgh, Pittsburgh, Pennsylvania, USA; 15 Surgery, University of Miami School of Medicine, Miami, Florida, USA; 16 Surgery, Yale University School of Medicine, New Haven, Connecticut, USA; 17 Surgery, Bridgeport Hospital, Bridgeport, Connecticut, USA; 18 University of Maryland Baltimore, Baltimore, Maryland, USA; 19 Neurosurgery, Indiana University School of Medicine, Indianapolis, Indiana, USA; 20 Emergency Medicine, University of Washington, Seattle, Washington, USA

**Keywords:** Efficiency, Research, Surveys And Questionnaires

## Abstract

**Background:**

Trauma remains one of the leading causes of death and disability in the USA, yet trauma research continues to suffer from inconsistent data collection standards, hindering data aggregation and interoperability. To advance trauma science and improve patient outcomes, the Department of Defense, in collaboration with the Coalition for National Trauma Research (CNTR), aimed to identify and implement a core set of common data elements (CDEs) for universal use in trauma research.

**Methods:**

A consensus-driven methodology guided by ACCORD reporting standards was employed. CNTR established a CDE Steering Committee comprising military, civilian, and academic trauma experts. Existing trauma and traumatic brain injury CDEs from the National Trauma Research Repository (NTRR) and the Federal Interagency Traumatic Brain Injury Research Informatics System were systematically compared, harmonized, and refined using informatics tools and expert feedback. The CDE Steering Committee met to evaluate 50 candidate data elements, with consensus defined as ≥80% agreement a priori.

**Results:**

Nineteen committee members (9 in person, 10 virtually) participated in the survey. A final set of 10 core trauma CDEs was approved for inclusion in NTRR v2, categorized under demographics and injury characteristics. An additional 22 elements were referred to domain-specific workgroups for consideration in future basic CDE development. This process marks a critical step in improving trauma data harmonization.

**Discussion:**

The adoption of these core trauma CDEs will support harmonization across trauma studies, enabling data reuse, pooled analysis, and cost-efficient trauma research. While consensus-based, this approach allows flexibility across study types but does not guarantee data harmonization. Successful implementation hinges on endorsement from funders and institutions. Future work includes developing therapeutic area-specific basic CDEs and promoting CDE adoption through training, technical support, and dissemination strategies.

**Level of evidence:**

Not applicable (methods-focused study).

**WHAT IS ALREADY KNOWN ON THIS TOPIC**

- There is limited ability to perform secondary analysis and combine studies in the National Trauma Research Repository (NTRR) due to a lack of standardized variable definitions and permissible values.

**WHAT THIS STUDY ADDS**

- This is a consensus-driven approach to creating standardized data elements, attributes, and values in the NTRR.

**HOW THIS STUDY MIGHT AFFECT RESEARCH, PRACTICE OR POLICY**

- This work provides basic common data elements for new trauma studies that will allow for secondary analyses of studies in the NTRR.

## Background

 In the USA, trauma accounts for approximately 41 million emergency department visits and 2 million hospital admissions annually. It claims more years of productive life lost than any other disease, including cancer. In 2023, trauma was estimated to have cost the nation more than $4.8 trillion in medical expenses and lost productivity.^1^ Despite this enormous societal burden, funding for trauma research from federal sources such as the National Institutes of Health (NIH) and the Department of Defense (DoD) significantly lags behind that of other diseases.^2^ Partly due to this scarcity of resources, critical knowledge gaps remain, contributing to the estimate that up to 20% of trauma-related deaths may be preventable with timely and informed interventions.^3^

To address these disparities and move toward the goal of zero preventable deaths, the 2016 NASEM report, “A National Trauma Care System: Integrating Military and Civilian Trauma Systems to Achieve Zero Preventable Deaths After Injury,” outlined 11 comprehensive recommendations for national trauma care reform.^3^ Among these recommendations was the development of common data standards and the creation of a system that enables military and civilian trauma systems to collect and share common data spanning the entire continuum of care.

Primary data collection involves obtaining information directly from the source, such as through surveys, clinical measurements, or interviews. Data abstraction refers to extracting information from existing records, including medical charts or registries. In trauma research, several national databases exist that allow for data abstraction, including the National Burn Data Standard, Spinal Cord Injury Model Systems, National Trauma Data Bank, National Trauma Research Repository (NTRR), and the Federal Interagency Traumatic Brain Injury Research (FITBIR). These resources offer rapid access to large datasets, reduce the cost and time associated with data collection, reflect real-world clinical practice rather than the controlled conditions of prospective studies, and enable exploratory analyses to guide future research. Currently, among these databases, there is a lack of standardization of common data elements (CDEs). A CDE is a standardized, precisely defined variable with a set of allowable responses used consistently across studies or sites.^4 5^

The DoD, through its partnership with the Coalition for National Trauma Research (CNTR), a research coordinating center comprising 25 trauma organizations, aimed to review and update existing CDEs for both trauma and traumatic brain injury (TBI) and develop a long-term support system to assist future trauma study submissions to the NTRR/FITBIR informatics system. FITBIR is a biomedical informatics system and data repository for TBI research that was developed through the collaborative effort of the NIH Institutes and Centers and the US Army Medical Research and Development Command.

The purpose of this project was to identify the core CDEs to be used in trauma studies funded through both civilian and military mechanisms in the USA and to integrate these to the extent possible with FITBIR. A modified Delphi method was chosen.

## Method

The DoD provided funding for this project to CNTR. CNTR employed a consensus-driven approach involving national and international trauma researchers to identify and specify a small set of core trauma CDEs to be collected in all trauma studies and prepare to integrate legacy trauma study data into the FITBIR entirely. This study followed the Accurate Consensus Reporting Document (ACCORD) guidelines for reporting consensus methods in biomedicine.^6^ The phases of identifying the core CDEs are reported below.

### Establishment of a steering committee

To guide this work, the NTRR Executive Steering Committee (comprising representatives from the Defense Health Agency’s (DHA) Research and Engineering (R&E), Combat Casualty Care (CCC) Portfolio, the National Institute of Neurological Disorders and Stroke (NINDS), and NIH Center for Information Technology (NIH-CIT)) established a CDE Steering Committee to identify a set of core CDEs recommended for collection in all trauma studies. The CDE Steering Committee included two individuals from the Multi-Institutional Trials Committees of several CNTR member organizations (American Association for the Surgery of Trauma (AAST), American Burn Association (ABA), Eastern Association for the Surgery of Trauma (EAST), and Western Trauma Association (WTA), the American College of Surgeons Committee on Trauma (ACS-COT), and an international representative who leads the National Trauma Quality Improvement Program at the ACS-COT. The CDE Steering Committee also included representatives from the American Association of Neurosurgical Surgeons (AANS), the Society of Critical Care Medicine (SCCM), the DHA R&E CCC, NIH, and NINDS, as well as subject matter experts in emergency medicine.

### Preparatory research

Working with a data curator at NIH-CIT, CNTR conducted a systematic comparison between the core CDEs from the original NTRR and the existing FITBIR core. This comparison aimed to eliminate duplicative elements and create a consolidated, refined list of core CDEs. Cross-mapping of NTRR core and FITBIR core CDEs was completed using a Python script developed by NIH-CIT to provide semantic and cross-encoder similarity scores. This analysis identified similar core CDEs within both data dictionaries and ensured that attributes such as variable name, title, definition, and permissible values aligned with current FITBIR data structures. In total, 30 FITBIR and 20 NTRR CDEs were evaluated. These results, along with the list of core CDEs from NTRR and FITBIR, and the attribute comparison of data structures, were uploaded to a page on the FITBIR website to inform the trauma research community and provide a means for public comment.

To encourage feedback, CNTR undertook a multichannel outreach campaign. CNTR created a NatTrauma.org home page banner that pointed visitors to a page describing the project and linking to the FITBIR page that contains the cross-mapping and attribute comparison. A feedback form on the page enabled those interested to provide comments. CNTR also created a postcard describing the call for feedback and distributed it at the AAST meeting in September 2023, and at the EAST meeting in January 2024. CNTR also provided the postcard in digital form to the Executive Steering Committee, the CDE Steering Committee, and the CNTR Board of Directors for distribution to colleagues and other organizations.

Subsequently, CNTR performed a literature review of FITBIR CDE development to identify previous methodologies used for CDE selection. Using this background, preliminary workplans were drafted to guide the selection of core CDEs by the CDE Steering Committee and sets of basic CDEs by several therapeutic area-specific workgroups. (Basic CDEs are those recommended to be collected in specific areas of care, not in all studies. See other manuscripts in this supplement for details.) CNTR’s data analyst met bi-weekly with NIH-CIT staff from mid-2023 to mid-2024 to refine the CDE selection protocol, discuss optimal data structures, and create the data classifications within the FITBIR system to match with each of the workgroups to be established (Epidemiology, Prehospital, Acute Hospital, and Rehabilitation/Outcomes).

To further refine the dataset, form administration data elements were automatically adopted in the NTRR. Form administration data elements are those that appear on form structures when data is collected to identify how forms were administered and to provide consistency and context for all data submissions. Forms include data elements describing such details as the language used on the form and who completed the form (patient, physician, spouse, etc.). FITBIR elements specific to TBI were excluded. The refined list of core CDEs (n=29) was then grouped into four different subject areas for ease of analysis (demographics, injury, medical history, and enrollment; see table 1). Finally, definitions from existing data elements in FITBIR and the American College of Surgeons National Trauma Data Standard (NTDS) were collected for a selection process during a 2-day CDE Steering Committee Meeting.

**Table 1:** Current Core CDEs in NTRR and FITBIR for consideration by the CDE Steering Committee

Current Core CDEs in NTRR and FITBIR	
Demographics	
Source	Data element
NTRR v1/NTDS	Age
FITBIR general core/NTDS	Birth date
FITBIR general core/NTRR v1/NTDS	Ethnicity
FITBIR general core/NTRR v1/NTDS	Race
FITBIR general core	Gender type
NTRR v1/NTDS	Person sex
FITBIR general core	Sex subject genotype type
FITBIR general core	Education school participation status
FITBIR general core	Employment status

Injury	
Source	Data element
NTRR v1/NTDS	AIS body region
NTRR v1/NTDS	AIS dictionary version type
NTRR v1/NTDS	AIS injury description text
NTRR v1/NTDS	AIS injury severity score
NTRR v1	AIS PreDot number
NTRR v1/NTDS	Injury severity score
NTRR v1/NTDS	Injury ICD E-code
NTRR v1	ICD version
NTRR v1/NTDS	Injury date time

Medical history	
Source	Data element
FITBIR general core/NTRR v1/NTDS	Medical history condition/comorbidity/pre-existing condition
FITBIR general core	Medical history condition SNOMED CT code
FITBIR general core	Medical history condition start date time
FITBIR general core	Medical history taken date time

Enrollment	
Source	Data element
FITBIR general core	Enrolled study indicator (enrolled yes/no)
FITBIR general core	Enrolled study date time
FITBIR general core	Informed consent obtained indicator (yes/no)
FITBIR general core	Informed consent obtained date time
FITBIR general core	Off study reason
FITBIR general core	Off study date time
FITBIR general core	Study eligibility indicator (yes/no)

AIS, Abbreviated Injury Scale; CDEs, common data elements; FITBIR, Federal Interagency Traumatic Brain Injury Research; ICD, International Classification of Diseases; NTDS, National Trauma Data Standard; NTRR, National Trauma Research Repository.

### Assessing consensus

During a 2-day meeting, the CDE Steering Committee engaged in a general discussion to ensure all participants clearly understood the task: to determine which of the existing trauma core CDEs should be retained and whether any additional core CDEs should be added to the NTRR moving forward. This discussion was moderated by staff from CNTR, DHA R&E CCC, and NIH, both in person and through Zoom. During this discussion, the group agreed to define consensus as ≥80% agreement.

Following this discussion, CNTR staff provided participants with a link to a prepared survey to systematically indicate whether each element should be classified as a core element, a basic element (relevant to one or more therapeutic environments and referred to those workgroups), or removed entirely. No pilot was conducted to evaluate the survey. The committee members who participated in the survey did not contribute to its design or development.

After survey completion, CNTR staff presented the aggregated results to the committee to ensure anonymity of responses. Using a directed consensus process, the committee reviewed the results. Each element was considered in the context of its definition and permissible values, allowing participants to refine their assessments further and determine whether any of the initially selected core CDEs should instead be assigned to a subsequent workgroup.

### Participation

Honoraria payments and travel reimbursements were provided to the CDE Steering Committee members. The surveys and the meetings were conducted in English.

### Reaching consensus on checklist items

The committee reviewed the core data elements that existed in FITBIR and NTRR. CNTR staff developed an online survey to facilitate the discussion, which enabled committee members—both in the room and attending remotely—to provide their input on the data elements being considered for the core. The initial study design was planned to conduct a Delphi-style evaluation of the data elements; however, due to the need for detailed discussion, the process was transitioned to an in-person consensus meeting. While the data elements themselves were generally agreed on, the in-person discussions were essential for clarifying definitions and permissible values, particularly where discrepancies existed between the NTRR and FITBIR databases. By thoroughly discussing each data element and permissible value list, the committee was able to arrive at consensus.

## Results

The 2-day meeting of the CDE Steering Committee was held 6–7 February 2024. There were nine members present and nine members who participated via Zoom. The survey was administered on the morning of 6 February. The response rate to the survey was 100%. An analysis was performed, and the results of the survey were reported on the afternoon of 6 February.

A directed discussion ensued as the results of the survey were displayed. After review and thorough discussion of each data element and permissible value list, the CDE Steering Committee arrived at consensus on a final set of 11 core data elements (table 2). The Core CDE related to Gender was subsequently removed to comply with a presidential executive order, for a total of 10 Core CDEs.^7^ The final list of 10 (11) CDEs was agreed on with 100% consensus among participants.

**Table 2:** Selected NTRR Core CDEs and definitions

Core CDEs for NTRR v2		
Data element	Definition	Permissible values
Age years	Value for participant’s subject age, calculated by elapsed time since the birth of the participant/subject in years to the injury.	
Ethnicity	Category of ethnicity the participant/subject most closely identifies with.	Hispanic or Latino | Not Hispanic or Latino | Not reported | Unknown
Race*	The subject self-declared racial origination, independent of ethnic origination, using Office of Management and Budget (OMB) approved categories.	American Indian or Alaska Native | Asian | Black or African American | Native Hawaiian or Other Pacific Islander | Not Reported | Unknown | White
Gender	On Day 2 of the meeting, Dr. McAuliffe suggested, and the group agreed, that the data element “Gender” is more up to date and should be recommended instead of “Gender Type.” Characteristics of people that are socially constructed, including norms, behaviors, and roles based on sex. As a social construct, gender varies from society to society and can change over time. (Adapted from WHO.)	Man | Woman | Non-Binary | Transgender | None of these describe me | I would like to consider different options | Prefer not to Answer
Sex at birth	On day 2, the group determined the “Sex at Birth” element was more appropriate than Person Sex. The physical sexual characteristics of the neonate at birth. A textual description of a person’s sex at birth.	Female | Male | Intersex | None of these describe me | Prefer not to Answer
AIS body region	Category of body region assessed by Abbreviated Injury Scale (AIS).	Abdomen | External | Face | Head | Lower Extremity, Pelvis and Buttocks | Neck | Other Trauma | Spine | Thorax | Upper Extremity
AIS dictionary version type	A type/version of the AIS dictionary used to assess the injury.	AIS Revision 1990 | AIS Revision 1990 Update 1998|AIS Revision 2005|AIS Revision 2005 Update 2008|AIS Revision 2015
AIS injury severity score	An injury severity score (AIS code), as part of AIS code.	1|2|3|4|5|6|9
AIS code	AIS code including pre-dot and post-dot numeric codes as recorded according to the specified version of AIS dictionary.	
Injury ICD E-code	Code to classify the external cause of the injury.	
ICD version	Number of the International Classification of Diseases (ICD) used to obtain the code.	ICD-10| ICD-11|ICD-9

CDEs, common data elements; NTRR, National Trauma Research Repository.

Through the course of the discussion, the committee determined that several data elements were important but not appropriate for the core, and they referred these to five Basic CDE workgroups for consideration (see box 1: elements referred to basic CDE workgroups). They also determined the scope of work for the workgroups and recommended subject-matter experts to serve on them. The committee determined that the workgroups would be established in four therapeutic areas: Epidemiology, Prehospital, Acute Hospital, and Outcomes/Rehabilitation. Through a general discussion of sex, gender, race, ethnicity, and other social determinants of health (SDOH), it was determined that a fifth workgroup should be established to address these data elements. This SDOH workgroup would commence following the completion of the work by the four initial workgroups, so the SDOH group would be able to review all the data elements being recommended to determine what gaps remained. Papers detailing the efforts of these workgroups and the basic CDEs approved for inclusion in the NTRR’s data dictionary are published in this issue.

Box 1 **Elements referred to basic common data element workgroups**

**Elements for the epidemiology workgroup:**

- Date of birth.
- Sex subject genotype type.
- Education school participation status.
- Employment status.
- Injury severity score.
- SNOMED CT code.
- Medical. History condition start date/time.

**Elements for the prehospital workgroup:**

- Injury date/time.
- Institution level/transfer/process.
- NEMSIS Universally Unique Identifiers.

**Elements for the acute hospital workgroup:**

- Complications.
- Pre-existing conditions/medical history.
- Trauma center level.
- Transfer process.
- Discharge date/time.

**Elements for the outcomes/rehabilitation workgroup:**

- Education participation (baseline).
- Discharge disposition.
- Mortality.
- Postdischarge transfer/process.

## Discussion

Advances in post-injury survival rates attributed to clinical trauma research have been accomplished primarily through separate and disconnected efforts. While dispersed clinical researchers may pursue similar lines of investigation, computer systems do not easily transmit, receive, combine, analyze, and use shared data. With primary data collection and chart reviews, clinical research data are fragmented, sometimes within one facility, and can rarely be repurposed to answer additional research questions. Even when funded through federal mechanisms, individual project data collection is not coordinated with other projects, resulting in duplications, inefficiencies, and increased costs. The ability to harmonize and share data maximizes its value, promotes the conduct of follow-up studies, and minimizes duplicative data collection. Recognizing these benefits, several research networks (including the NIH and PCORnet) have promoted data standardization and the use of CDEs to improve interoperability across studies.^8 9^

Additionally, trauma studies often have small sample sizes with limited statistical power to evaluate interventions due to the difficulty in obtaining informed consent from trauma patients or their legally authorized representatives. However, pooled datasets (from multiple studies using CDEs) can provide the additional statistical power necessary to demonstrate clinical and statistical significance. Thus, the ability to make aggregated research data and larger samples widely available to clinical investigators is critical to advancing trauma research and care. Many federal funding agencies are now mandating that results from funded studies be shared. Data sharing is actively encouraged by initiatives such as the SCCM’s Data Science Campaign, which has published a set of guiding principles developed through a systematic literature review and a modified Delphi process. These principles aim to enhance data accessibility, standardization, and interoperability across critical care settings.^10^

The NTRR is a collaborative initiative involving the CNTR, the DoD, and the NIH.^11^ Hosted by the NIH and funded by the DoD, the NTRR serves as the central repository for clinical trauma research data generated from both military and civilian studies, regardless of whether they are federally or privately funded. It was developed to meet the growing need for data sharing within the trauma research community and is intended to serve as a foundational component of the national trauma research infrastructure. Adoption of core and basic CDEs will improve the interoperability of clinical trauma data, allowing investigators to share study manuals, data dictionaries, publications, and study metadata with other investigators so that they can perform secondary or aggregate analyses.

The strength of this study lies in the collective opinion of experts in the field, with an initial anonymous survey that provided space for reflection and refinement of ideas. The modified aspect of this study was the second consensus step, which involved an in-person meeting to finalize the CDEs. While the data elements themselves were generally agreed on, the meeting was essential for clarifying the definitions and permissible values for each data element, especially due to the discrepancies between the NTRR and FITBIR related to the permissible values of several data elements. Although this in-person meeting impacted the anonymity of interactions, it was crucial for quickly reaching consensus on the finer details that would have been difficult to achieve through a survey alone.

Additionally, it is essential to acknowledge the limitations of our approach to defining a CDE as an underlying concept. By defining the CDE as the underlying concept (eg, hemoglobin) and treating modifiers (eg, time of collection) separately, we promote interoperability but do not guarantee that data can be aggregated seamlessly across all studies. This flexible structure, while departing from more rigid data models, offers distinct advantages.^12^ It enhances the utility of CDEs, supports a broader range of study designs, and improves adoption and compliance. However, this flexibility comes with trade-offs, and the approach should be understood as a step toward greater harmonization, rather than a complete solution. Additionally, widespread adoption of these CDEs by the trauma research community is not guaranteed unless their use is supported, if not mandated, by major funding agencies or influential professional organizations. Without such endorsement, the NTRR data dictionary may not achieve its intended impact as a standard for data collection and interoperability.

The output of this CDE process is a critical next step in enabling practical evaluation of trauma studies and facilitating the development of future research by creating an agreed-on data dictionary that defines core elements with standardized, consensus-based definitions. Establishing such a dictionary enables data collected across studies to be comparable and suitable for aggregation—laying the foundation for robust, multicenter analyses and long-term knowledge generation in trauma research.

In the next phase of the NTRR project, supported with an additional 2 years of DHA funding, CNTR is working on implementation. Efforts to inform the broader trauma research community about the utility of the NTRR are underway, including surgery podcast episodes and public service announcements; exhibitions, posters, and presentations at major scientific conferences; quarterly webinars targeted to specific user groups; and monthly advertisements on CNTR media platforms. The CNTR team is offering general support for uploading data from completed studies and aligning researchers’ new study designs with the NTRR core and basic CDEs. As a subawardee on a University of Chicago project titled *Neurotrauma Training Program for Military Providers: Bridging Gaps in Deployed Neurosurgical Capability and Improving Wartime Readiness*, CNTR will be convening a workgroup of subject matter experts to develop basic CDEs and standard terminology for penetrating TBI studies. Finally, as meta studies are created within the NTRR, CNTR will work with NIH-CIT to assess whether new CDEs should be developed to fulfill emerging needs and whether unique data elements are being used frequently enough to be officially designated as CDEs.

## Data Availability

Data sharing not applicable as no datasets generated and/or analyzed for this study.
